# Effects of altered ephrin-A5 and EphA4/EphA7 expression on tumor growth in a medulloblastoma mouse model

**DOI:** 10.1186/s13045-015-0202-9

**Published:** 2015-09-07

**Authors:** Shilpa Bhatia, Kellen Hirsch, Nimrah A. Baig, Olga Rodriguez, Olga Timofeeva, Kevin Kavanagh, Yi Chien Lee, Xiao-Jing Wang, Christopher Albanese, Sana D. Karam

**Affiliations:** Present address: Department of Radiation Oncology, University of Colorado Denver, Anschutz Medical Campus, Aurora, CO 80045 USA; Department of Oncology, Lombardi Comprehensive Cancer Center, Georgetown University Medical Center, Washington, DC 20057 USA; Department of Pathology, University of Colorado Denver, Anschutz Medical Campus, Aurora, CO 80045 USA; Department of Pathology, Georgetown University School of Medicine, Washington, DC 20057 USA

## Abstract

**Background:**

Members of the Eph/ephrin gene families act as key regulators of cerebellar development during embryogenesis. Aberrant signaling of Eph family of receptor tyrosine kinases and their ephrin ligands has also been implicated in human cancers. Medulloblastoma is an aggressive primitive neuroectodermal tumor that originates from granule neuron precursors in the cerebellum. Previous studies have suggested a role for the ephrin-A5 ligand and its receptors, EphA4 and EphA7, in granule cell-precursor formation and in guiding cell migration. In the present study, we investigated the effects of genetic loss of ephrin-A5, EphA4, and EphA7 on the spatiotemporal development of medulloblastoma tumors in the context of the smoothened transgenic mouse model system.

**Findings:**

Radiographic magnetic resonance imaging (MRI) was performed to monitor tumor growth in a genetically engineered mouse model of medulloblastoma. Tumor tissue was harvested to determine changes in the expression of phosphorylated Akt by Western blotting. This helped to establish a correlation between genotype and/or tumor size and survival. Our in vivo data establish that in ND2-SmoA1 transgenic mice, the homozygous deletion of *ephrin-A5* resulted in a consistent pattern of tumor growth inhibition compared to their *ephrin-A5* wild-type littermate controls, while the loss of *EphA4/EphA7* failed to produce consistent effects versus *EphA4/EphA7* wild-type mice. A positive correlation was evident between tumor size, p-Akt, and proliferating cell nuclear antigen (PCNA) expression in our transgenic mouse model system, regardless of genotype.

**Conclusions:**

Taken together, our findings underscore the importance of targeting specific members of the Eph/ephrin families in conjunction with the Akt pathway in order to inhibit medulloblastoma tumor growth and progression.

## Findings

### Introduction

The Eph family of receptors and their cognate ephrin ligands have been implicated in processes occurring during embryonic development [1]. Emerging evidence suggests a role for Eph/ephrin axes in tumor cell proliferation, survival, migration, and angiogenesis [1]. Medulloblastoma is a pediatric brain tumor that arises from granule neuron precursors in the cerebellum [2]. Previous studies have reported that ephrin-A5 and its high-affinity binding receptors, EphA4 and EphA7, play a key role in granule cell-precursor formation and migration from the external granule cell layer to the internal granule cell layer [3, 4].

In the present study, we sought to determine the effects of loss of ephrin-A5 and the EphA4/EphA7 receptor pair on medulloblastoma tumor growth in vivo. The mouse models were generated by breeding the ND2-SmoA1 mice (Fred Hutchinson Cancer Research Center, Seattle, WA, USA) used in previous studies [5–8] with *ephrin-A5*^*−/−*^ mice or *EphA4*^*−/−*^*EphA7*^*−/−*^ mice. The ND2-SmoA1 model expresses a constitutively activated form of the Smoothened (SmoA1) gene that is expressed in cerebellar granule cell precursors under regulation of the NeuroD2 (ND2) promoter [9]. Activated Smoothened present on granule cell precursors interacts with the sonic hedgehog (SHH) receptor present on Purkinje cells, resulting in continuous proliferation of granule cell precursors. Using magnetic resonance imaging (MRI)-based longitudinal imaging approach, we investigated whether knockout of ephrin-A5 (present on granule cell precursors [3]) and its receptors, EphA4/EphA7 (present on Purkinje cells [3]), would interfere with the spatiotemporal development of tumors in this model system. A correlation was observed between genotype and tumor size in ephrin-A5 knockout mice with loss of ephrin-A5 resulting in smaller tumors compared to wild-type littermate controls. This correlation, however, was not consistently observed in the EphA4/EphA7 double-knockout mice. Previous studies have reported SHH-induced medulloblastoma formation in mouse models via activation of the Akt signaling pathway through Akt phosphorylation [2]. When examining the effects of PI3K/Akt inhibitor in medulloblastoma cell lines, Hartmann et al. [10] showed that it is proliferation and not apoptosis that was dependent on PI3K/AKT pathway. Considering this, we analyzed the expression of p-AKT, AKT, and its downstream molecule proliferating cell nuclear antigen (PCNA), a key player in cancer cell proliferation, in the tumor tissues. Regardless of genotype, a correlation between tumor size and p-Akt and PCNA was observed, with larger tumors (>400 mm^3^) possessing higher relative levels of p-Akt and PCNA versus smaller tumors.

## Material and methods

### Generation of *ephrin-A5*^*−/−*^*Smo* mice

An *ephrin-A5*^*−/−*^*Smo* medulloblastoma mouse model was generated by breeding the ND2-SmoA1 mouse model [11] (gift from Dr. James Olson, Fred Hutchinson Cancer Research Center, Seattle, WA, USA) to the *ephrin-A5*^*−/−*^ mice. The *ephrin-A5*^*−/−*^ mice were obtained from the Jackson Laboratory (Bar Harbor, ME). All mice were kept, handled, and euthanized in accordance with the ethics guidelines set and overseen by the Georgetown University Animal Care and Use Committee. Mice were genotyped and the presence of the SmoA1 transgene cassette was verified by PCR using a standard protocol [11]. The *ephrin-A5*^*+/+*^*Smo* mice with wild-type expression of the ephrin-A5 gene were used as controls. A total of 40 mice were included in the study. For the most part of the analysis, only littermate- and gender-matched controls were used. A minimum of three to four mice were included in each experimental/control group.

### Generation of *EphA4*^*−/−*^*EphA7*^*−/−*^Smo mice

An *EphA4*^*−/−*^*EphA7*^*−/−*^*Smo* medulloblastoma mouse model was generated by breeding the ND2-SmoA1 mouse model [11] to the *EphA4*^*−/−*^*EphA7*^*−/−*^ mice [12]. The *EphA4*^*−/−*^*EphA7*^*−/−*^ double-knockout mice (gift from Dr. Maria Donoghue, Georgetown University, Washington, DC, USA) were generated as previously described [12].

Attempts at generating EphA7 SmoA1 mice failed. EphA7^*−*/*−*^ mice are known to display phenotypes of hyperactivity and aggressiveness similar to what has been previously described with other ephrin knockout mice [13]. Additionally, EphA7^−/−^ mice become obese as they age, making it technically quite challenging to mate them to a level sufficient to generate a genetically engineered animal model. Therefore, we initiated breeding on EphA4/EphA7 double-knockout background as surprisingly their behavior was tamer on that background and mating was not an issue. Most importantly, EphA4 serves as the second high-affinity binding receptor to the ephrin-A5 ligand. By knocking down both of ephrin-A5’s highest-affinity binding receptors, we thought to gain a better understanding of functional inhibition of this pathway.

Genotyping and PCR analysis was performed using an established protocol [11, 14]. The *EphA4*^*−/−*^*EphA7*^*−/−*^*Smo* model therefore lacks both EphA4 and EphA7 gene expression and expresses a constitutively activated form of Smoothened (SmoA1) gene in cerebellar granule precursor cells. The *EphA4*^*+/+*^*EphA7*^*−/−*^*Smo* mice were used as controls. A total of 37 mice were included in the study. For the most part of the analysis, only littermate- and gender-matched controls were used. A minimum of two and maximum of four mice were included in each experimental/control group.

### Magnetic resonance imaging

MRI was performed to monitor tumor development in mice [6, 14] using the 7T Bruker BioSpec Avance III horizontal magnet in the Georgetown-Lombardi Preclinical Imaging Research Laboratory. Volumetric analysis was done on the images using the following formula: [(longest diameter)*(shortest diameter)^2^]/2. Tumors >400 mm^3^ were considered to be large in size and tumors <10 mm^3^ were considered to be small in size. Statistical analysis was done using Student’s *t* test. *p* ≤ 0.05 was considered significant.

### Western blotting

Tumor tissue lysates were prepared for analysis of protein expression by Western blotting using a standard protocol [15]. The primary antibodies (anti-p-AKT, AKT, and anti-β-actin) were obtained from Cell Signaling Technology (Danvers, MA, USA). PCNA antibody was obtained from BD Biosciences (San Jose, CA, USA). Horseradish peroxidase (HRP)-conjugated secondary antibodies were obtained from Sigma (St. Louis, MO, USA). Western analysis on the molecular markers was repeated two to three times. Densitometric analysis was done on the images using Image J software. p-Akt levels were normalized to total Akt and PCNA levels were normalized to β-actin. Statistical analysis was done using Student’s t test. *p* ≤ 0.05 was considered significant.

## Results

### Genetic loss of ephrin-A5 inhibits tumor growth in the Smo/Smo mouse medulloblastoma model and affects Akt phosphorylation and PCNA expression

**Fig. 1:**
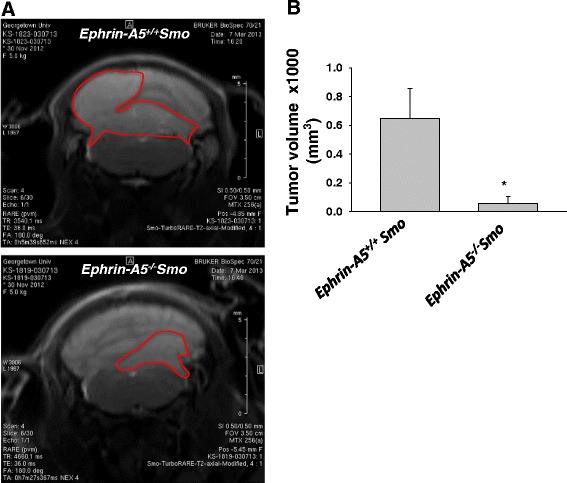
MRI-based morphometric analyses identified a reduction in tumor size in the *ephrin-A5* ^*−/−*^ *Smo mouse* model. **a** Representative images are shown for *ephrin-A5* ^*−/−*^ *Smo* mice and their *ephrin-A5* ^*+/+*^ *Smo* littermate controls, monitored by sequential MRI. **b** Volumetric tumor growth analysis. Data represent mean ± standard deviation from different tumor tissues (*p ≤ 0.05)

**Fig. 2:**
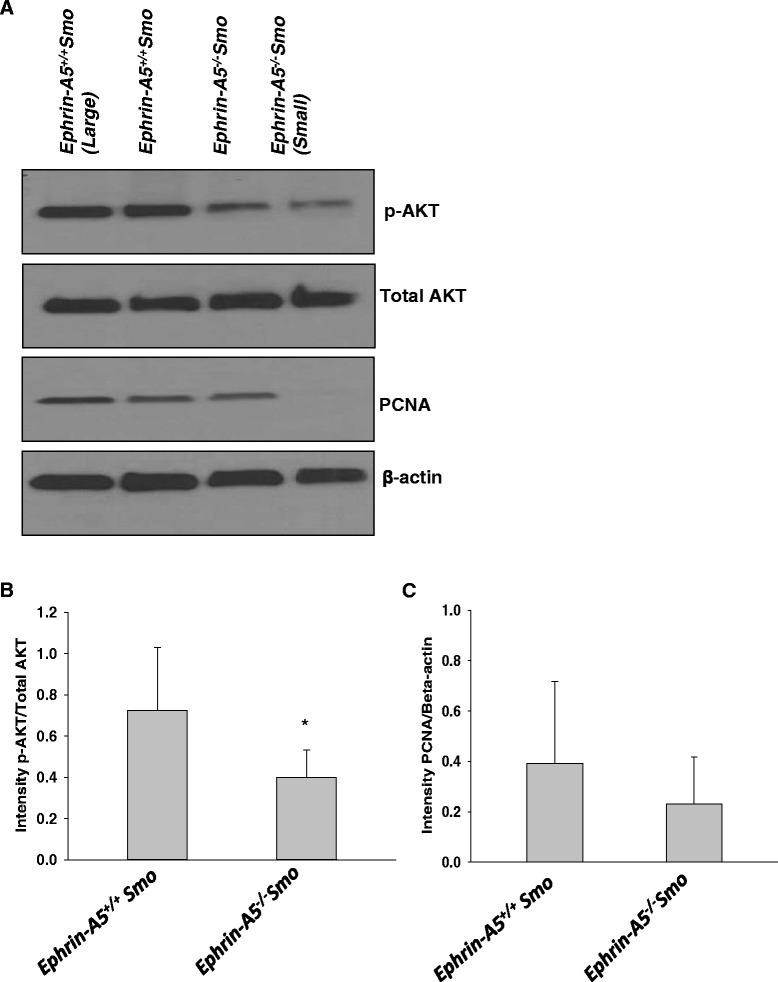
Western blot analysis shows altered expression of p-Akt and PCNA that related to tumor size and genotype in *ephrin-A5* ^*−/−*^ *Smo mouse* model. **a** Western blot analysis in representative tumor sections suggests a decrease in p-Akt and PCNA expression in smaller tumors (<10 mm^3^) with loss of the ephrin-A5 gene. Total Akt levels seem to be unchanged. **b** Densitometric analysis of p-Akt expression (*p ≤ 0.05) and **c** PCNA expression (*p* = not significant) in *ephrin-A5* ^*+/+*^ *Smo* versus *ephrin-A5* ^*−/−*^ *Smo* tumors. Data represent mean ± standard deviation from different tumor tissues

To investigate the effect of loss of function of ephrin-A5 on medulloblastoma tumor growth in vivo, an *ephrin-A5*^*−/−*^*Smo* model was generated which lacks the *ephrin-A5* gene and expresses a constitutively activated form of Smoothened (SmoA1) gene in cerebellar granule precursor cells. MR imaging revealed reduced tumor growth in *ephrin-A5*^*−/−*^*Smo* mice compared to the wild-type *ephrin-A5*^*+/+*^*Smo* control mice (Fig. 1a, b). Furthermore, Western blot analysis performed on tumor tissue derived from ephrin-A5 knockout mice and their littermate controls established that the tumors from *ephrin-A5* knockout mice, which were smaller than in the *ephrin-A5* wild-type mice, also had significantly lower levels of phosphorylated Akt (Fig. 2a, b). Decreased PCNA expression was also evident in *ephrin-A5* knockout mice compared to *ephrin-A5* wild-type mice. However, the difference was not statistically significant (Fig. 2a, c). Total Akt levels seem to be unaltered (Fig. 2a).

Genetic alteration of EphA4/EphA7 yields no consistent effect on tumor size in the Smo/Smo mouse medulloblastoma model. However, correlation between tumor size and p-Akt levels as well as PCNA persists independent of genotype.

**Fig. 3:**
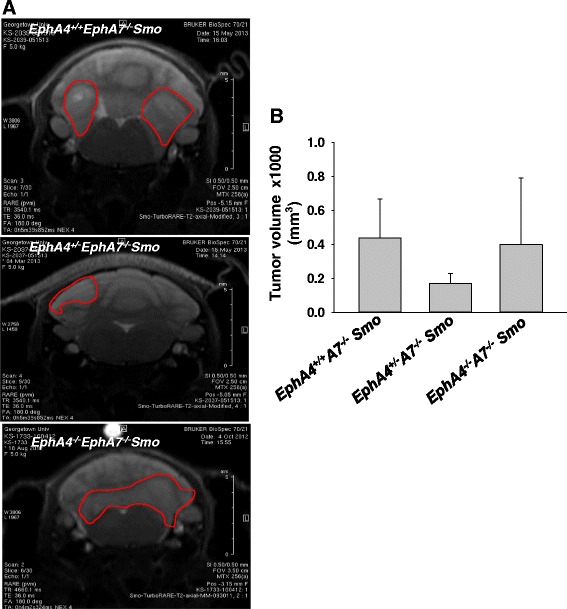
Representative MRI scans show that genetic alteration of EphA4/EphA7 genotype yields no consistent effect on tumor size in the Smo medulloblastoma animal model. *EphA4* ^*−/−*^ *EphA7* ^*−/−*^ *Smo* mice and their *EphA4* ^*+/−*^ *EphA7* ^*−/−*^ *Smo* and *EphA4* ^*+/+*^ *EphA7* ^*−/−*^ *Smo* littermate controls were followed by serial MRI. Representative images are shown for **a** *EphA4* ^*+/+*^ *EphA7* ^*−/−*^ *Smo*, *EphA4* ^*+/−*^ *EphA7* ^*−/−*^ *Smo*, and *EphA4* ^*+/−*^ *EphA7* ^*−/−*^ *Smo* pairs. **b** Volumetric tumor growth analysis. Data represent mean ± standard deviation from different tumor tissues. (*p* = not significant)

To determine the role of EphA4/EphA7 receptors on medulloblastoma tumor growth, sequential MRI was performed. Mice from different cohorts (*EphA4*^*−/−*^*EphA7*^*−/−*^*Smo*, *EphA4*^*+/−*^*EphA7*^*−/−*^*Smo*, and *EphA4*^*+/+*^*EphA7*^*−/−*^*Smo*) were examined. Imaging analyses were performed, and while some *EphA4*^*−/−*^*EphA7*^*−/−*^*Smo* mice had smaller tumors when compared to *EphA4*^*+/+*^*EphA7*^*−/−*^*Smo* and *EphA4*^*+/−*^*EphA7*^*−/−*^*Smo* mice, no consistent trend was observed (Fig. 3a, b).

**Fig. 4:**
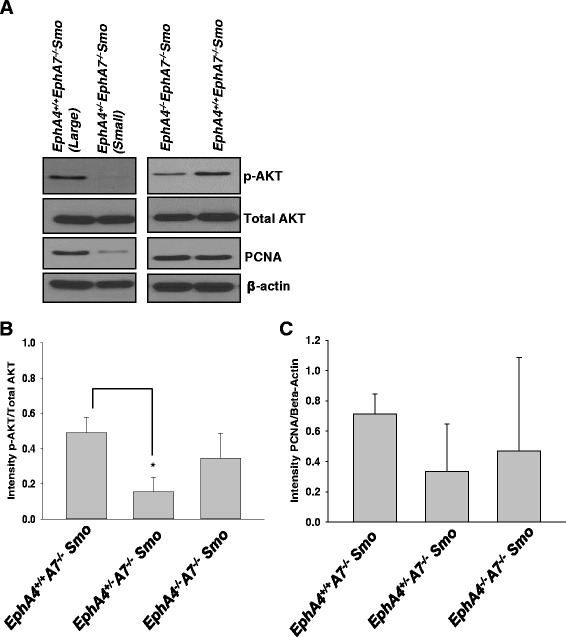
Western blot analysis suggests a correlation between p-Akt and PCNA levels and tumor size regardless of genotype in *EphA4* ^*+/+*^ *EphA7* ^*−/−*^ *Smo*, *EphA4* ^*+/−*^ *EphA7* ^*−/−*^ *Smo*, and *EphA4* ^*−/−*^ *EphA7* ^*−/−*^ *Smo* mice. **a** Western blot analysis in tumor tissues indicated expression of p-Akt and PCNA correlative of tumor size. Lysates were collected from mice and Western blot analysis was done to determine the expression of p-Akt, total Akt, and PCNA. **b** Densitometric analysis of p-Akt expression (*p ≤ 0.05) and **c** PCNA levels (*p* = not significant) in *EphA4* ^*+/+*^ *EphA7* ^*−/−*^ *Smo*, *EphA4* ^*+/−*^ *EphA7* ^*−/−*^ *Smo*, and *EphA4* ^*−/−*^ *EphA7* ^*−/−*^ *Smo* tumors. Data represent mean ± standard deviation from different tumor tissues

However, when p-Akt levels were examined across the different tumors here, we again found a correlation between tumor size and p-Akt levels irrespective of genotype within this subgroup. Western blot analysis of tumor tissue harvested from *EphA4*^*−/−*^*EphA7*^*−/−*^*Smo*, *EphA4*^*+/−*^*EphA7*^*−/−*^*Smo*, and *EphA4*^*+/+*^*EphA7*^*−/−*^*Smo* mice revealed expression of p-Akt (Fig. 4a, b) and PCNA (Fig. 4a, c) that correlated with actual tumor size. Total Akt levels seem to remain unchanged (Fig. 4a). In conclusion, although the combined loss of EphA4 and EphA7 receptors failed to show consistent effects on tumor growth, the correlation between levels of p-Akt, PCNA, and tumor size persisted.

## Discussion

Previous studies have demonstrated the importance of Eph receptors and their ephrin ligands in both early neural development and tumorigenesis [1, 16]. Specifically, ephrin-A5 has been shown to drive oncogenic potential in murine fibroblasts [17]. The role of ephrin-A5 in cancerous and normal neural development makes it a likely contributor to the growth and development of medulloblastoma as well.

The present study is the first to examine the impact of loss of ephrin-A5 on tumor size in medulloblastoma in vivo. Through MRI and tumor dissection, we examined a correlation between ephrin-A5 expression and medulloblastoma tumor size. Our findings reveal that the *ephrin-A5*^*+/+*^*Smo* mouse genotype correlates with larger tumors, while the *ephrin-A5*^*−/−*^*Smo* genotype correlates with smaller tumors. Additionally, we observed that the heterogeneous loss of *ephrin-A5* in the *ephrin-A5*^*+/−*^*Smo* genotype failed to bring about a statistically significant decrease in tumor size as compared to *ephrin-A5*^*+/+*^*Smo* controls (data not shown), suggesting that total ablation of ephrin-A5 expression is necessary for tumor growth inhibition. While limited by sample size and variations between animals, these data support the potential role of ephrin-A5 in tumor growth.

In the developing neural tube, a correlation between Smo activation and ephrin-A5 has been previously shown [18]. In the neural tube of E12-E14 mice and chick embryos, Hynes et al. [18] had shown that constitutive activation of Smo resulted in suppression of ephrin-A5 expression in the dorsal midbrain and hindbrain regions where Smo was overexpressed. In the developing cerebellum, we have previously shown that ephrin-A5 expressed in the external granule cell layers (EGL), which are medulloblastoma precursors [3]. Deregulated Smo expression in the EGL is one of mechanisms of medulloblastoma development. Our results suggest that Smo might be acting on enhancing granule cell proliferation via ephrin-A5 signaling. However, gain of function and loss of function experiments are needed before such hypothesis is validated.

Furthermore, since a constitutively active form of Smoothened mimics concentration-dependent actions of SHH [16], it would be of clinical interest to examine whether dual targeted inhibition of the SHH and ephrin-A5 impact medulloblastoma development.

Unlike the ephrin-A5 mice, data for the EphA4/EphA7 Smo mice did not yield any consistent patterns of variability in tumor size. This is likely related to a compensatory effect in response to a double knockout of ephrin-A5’s high-affinity binding receptors. This phenotypic robustness is likely due to functional redundancy by different members of the Eph gene family. EphA4 and EphA7 represent high-affinity binding ligands to the ephrin A5 ligands and are ubiquitously expressed in the cerebellum [3, 4]. However, ephrin-A5 is a promiscuous ligand that binds to multiple other Eph A and B receptors including EphB1 and EphB2 [19], both of which are present in medulloblastoma tumors [12, 20]. Further work is needed to understand the functional interactions between ephrinA5 and its receptors in vivo.

Another mechanism by which Smo exerts its effect on tumor growth is likely mediated *via* Akt pathway. Our data support a link between PI3K/Akt signaling and tumor size. Our data also support a direct correlation between tumor size, phosphorylation of Akt, and PCNA suggestive of a direct effect on cellular proliferation. The phosphoinositide-3-kinase and protein kinase B (PI3K/Akt) pathway has been implicated in many cancer types, including medulloblastoma [10, 21]. Phosphatidylinositol-3,4,5-triphosphate plays a critical role in recruiting Akt/protein kinase B to the plasma membrane, where it is phosphorylated by 3-phosphoinositol-dependent protein kinases [10]. Consequently, Akt phosphorylates various growth-effector molecules affecting cell growth, proliferation, and survival [10]. Phosphorylation and subsequent activation of Akt has been shown to correlate with medulloblastoma tumor growth and metastasis [22]. Indeed, resistance to hedgehog inhibition has been shown to be mediated via inappropriate activation of the PI3K pathway. Studies targeting the regulator of PI3K, PTEN—whose loss is common in medulloblastoma—have shown innate resistance to SHH inhibitors in PTEN null medulloblastomas compared to PTEN wild-type medulloblastoma [23]. Furthermore, PI3K/p-Akt has been shown to mediate medulloblastoma cell growth in a manner that is dependent on PTEN dysregulation [10]. Thus, combined targeting the PI3K/Akt pathway represents a rational approach to inhibit tumor growth and proliferation. Several preclinical and clinical studies have evaluated the safety and efficacy of therapeutic agents targeting this pathway [24–26]. Baryawno et al. demonstrated that blocking the PI3K/Akt pathway via inhibition of upstream PDK1 dramatically decreased tumor growth in medulloblastoma mouse models [27].

Taken together, these findings provide new insights into the potential treatment of medulloblastoma. Our established correlation between p-Akt and tumor size emphasizes the therapeutic relevance for PI3K/Akt pathway inhibitors for medulloblastoma treatment. Additionally, our findings suggest possible future therapeutic target for ephrin-A5 in medulloblastoma. These data, however, are limited by small sample sizes and the inherent biases and variations present in animal studies. Further studies are needed to explore the role ephrin-A5 might play in tumorigenesis of medulloblastoma.
